# Construction and Analysis of Emotion Recognition and Psychotherapy System of College Students under Convolutional Neural Network and Interactive Technology

**DOI:** 10.1155/2022/5993839

**Published:** 2022-09-17

**Authors:** Minwei Chen, Xiaojun Liang, Yi Xu

**Affiliations:** ^1^College of Physical Education, Chongqing University, Chongqing 400044, China; ^2^College of Humanities, Zhaoqing Medical College, Zhaoqing 526020, China; ^3^Ministry of Basic Education, GuangdongEco-Engineering Polytechnic, Guangzhou 510520, China

## Abstract

This study's aim is to effectively establish a psychological intervention and treatment system for college students and discover and correct their psychological problems encountered in a timely manner. From the perspectives of pedagogy and psychology, the college students majoring in physical education are selected as the research objects, and an interactive college student emotion recognition and psychological intervention system is established based on convolutional neural network (CNN). The system takes face recognition as the data source, adopts feature recognition algorithms to effectively classify the different students, and designs a psychological intervention platform based on interactive technology, and it is compared with existing systems and models to further verify its effectiveness. The results show that the deep learning CNN has better ability to recognize student emotions than backpropagation neural network (BPNN) and decision tree (DT) algorithm. The recognition accuracy (ACC) can be as high as 89.32%. Support vector machine (SVM) algorithm is adopted to classify the emotions, and the recognition ACC is increased by 20%. When the system's *K* value is 5 and *d* value is 8, the ACC of the model can reach 92.35%. The use of this system for psychotherapy has a significant effect, and 45% of the students are very satisfied with the human-computer interaction of the system. This study aims to guess the psychology of students through emotion recognition and reduce human participation based on the human-computer interaction, which can provide a new research idea for college psychotherapy. At present, the mental health problems of college students cannot be ignored; especially every year, there will be news reports of college students' extreme behaviors due to depression and other psychological problems. An interactive college student emotion recognition and psychological intervention system based on convolutional neural network (CNN) is established. This system uses face recognition as the basic support technology and uses feature recognition algorithms to effectively classify different students. An interaction technology-based psychological intervention platform is designed and compared with existing systems and models to further verify the effectiveness of the proposed system. The results show that deep learning has better student emotion recognition ability than backpropagation neural network (BPNN) and decision tree algorithm. The recognition accuracy is up to 89.32%. Support vector machine algorithm is employed to classify emotions, and the recognition acceptability rate increases by 20%. When *K* is 5 and *d* is 8, the acceptability rate of the model can reach 92.35%. The effect of this system in psychotherapy is remarkable, and 45% of students are very satisfied with the human-computer interaction of this system. This work aims to speculate students' psychology through emotion recognition, reduce people's participation via human-computer interaction, and provide a new research idea for university psychotherapy.

## 1. Introduction

With the rapid economic development, the society has undergone major changes, and various pressures such as life, employment, development, and learning have followed one another [1]. College is equivalent to half of society. As the main force of the next generation of society, college students are also faced with social, graduation, study, and life problems. These pressures invisibly bring serious mental obstacles to the positive development of students [2]. Thus, the importance of mental education and mental consultation in colleges and universities has become increasingly prominent [3]. Studies have shown that 23% of students in Chinese colleges and universities have different levels of mental problems, which proves that the problem of mental health of college students in China is becoming more serious. Therefore, timely research on the mental health of college students is of great significance for talent training and social development [4]. United Nations (UN) experts have predicted that “from the 21^st^ century, mental problems will be the chief culprit that plagues people's development and causes many other problems” [5]. The World Health Organization (WHO) has estimated that six of the top 20 major diseases will be mental diseases by 2020, accounting for 17.4% of all diseases [6]. As far as the level of mental health teaching in Chinese universities is concerned, some students with mental problems are unable to receive effective mental counseling and treatment in time due to the lack of mental counselors, which in turn leads to more students jumping off buildings and committing suicide [7]. Therefore, how to scientifically and effectively solve the shortcomings in mental counseling and treatment in colleges and universities has become the core issue discussed in the current mental education field.

Early warning of mental health is to timely discover and identify the factors affecting the mental health crisis through the identification and dynamic detection of the early warning object based on the analysis and processing of the object's behavior, emotions, and other data, so as to take timely preventive measures to reduce the more serious consequences caused by mental problems [8]. Therefore, it is widely used in various universities and enterprises. The early warning of mental health for students in colleges and universities is mainly realized through the relationship of student dormitory, class atmosphere, college system, and school pressure [9]. Wang et al. investigated on the mental health of some patients with diseases, revealing the protective factors related to the risk reduction of mental health problems in patients with diseases. This work revealed the high incidence of mental health problems among patients with diseases and the gap in mental health services, which also indicates the high degree of pain caused by the risk of disease environment and increased stress [10]. Hong et al. examined the mediating role of rumination and the moderating role of mindfulness in the association between social media exposure and COVID-19 information and psychological distress. A questionnaire survey was conducted among local college students to gain a better understanding of the development of psychological symptoms during the COVID-19 pandemic and provide insights into effective interventions for adverse mental health outcomes among college students [11]. Individuals and groups with abnormal psychology are screened, the student groups that may have mental problems are found and analyzed, the early warning messages are given based on their recent behaviors, and their behaviors are evaluated and analyzed by the mental processing centers of universities to achieve effective prevention of mental health crises [12]. Being different from students in other disciplines, physical education students suffer from greater employment restrictions, so they are more likely to have mental problems [13]. At present, early warning system of mental health in college often suffers from high cost, poor predictive performance, and more complicated system, which greatly hinder the development of college mental health education, making it difficult for early warning system of mental health in college to be applied in more colleges and universities [14].

In order to effectively provide early warning of mental health for physical education students and prevent college students from greater tragedies due to mental problems, a mental health prediction and treatment system for contemporary college students is constructed in this study starting with the existing problems of the existing college mental health early warning system and using the big data and artificial intelligence technology. Through data analysis, a database under different mental conditions is established, which can achieve effective human-computer interaction with the help of data. Therefore, this study can provide new research ideas and methods for mental health education in colleges. Firstly, the concept of deep learning-based emotion recognition algorithm is introduced, and the methods and measures of feature extraction of mental health data are explained. Secondly, the technical strategy of psychotherapy based on artificial intelligence is illustrated, and data training and parameter optimization are explained. Finally, the established model is employed to conduct performance evaluation and interaction analysis on the psychological parameters of college students to obtain the research results, and then the conclusion is drawn. This investigation provides some reference theory for college students' mental health diagnosis and treatment system. The innovation lies in the introduction of a psychological feature extraction method as the main basis of mental health judgment.

## 2. Literature Review

As places for talent training, colleges and universities have to understand the critical significance of big data to the mental health work [15]. Mental health educators in colleges and universities also need to develop the habit of emphasizing data investigation and forming data thinking and collect more relevant data through mental consultation and mental teaching of students [16]. There are many related studies on big data and mental health. Kulesza et al. used social media big data, natural language processing, and machine learning techniques to achieve mental health monitoring [17]. Huang et al. established a multiple regression model to examine the combined effects of fear and collectivism on the public's prevention intentions for COVID-19. The simple slope test was adopted to examine the interaction between fear and collectivism on preventive intention. It was found that fear and collectivism reduced the positive impact of each other on people's preventive intention [18]. Simon used big data to study the relationship between genome and mental health, which closely linked the genetic inheritance and mental health [19]. Thus, it can be concluded that the use of big data analysis methods can help people deeply analyze the behavior and thinking of the human body, establish a mental health knowledge system, and provide a basis for establishing an early warning system of mental health.

In recent years, with the continuous in-depth research and rapid development as well as the application of expert systems in the field of medical diagnosis, a good foundation has been laid for the development of intelligent mental consultation [20]. The intelligent auxiliary diagnosis system can give full play to the role of domain experts in diagnosis through the effective acquisition, transmission, processing, and utilization of diagnosis information [21, 22]. There are many related research studies on artificial intelligence and mental health. D'Alfonso et al. enhanced the user participation with computing and artificial intelligence methods and further modified the system and improved the ability of young people to cope with the mental stress with novel mechanisms [23]. Graham et al. discussed in detail the various applications of artificial intelligence in mental health, further proving the potential of applying machine learning algorithms to solve mental health problems [24]. Kalmady et al. found that machine learning techniques can be well applied to mental health analysis research based on the combination of machine learning and functional magnetic resonance imaging system [25]. Graham et al. demonstrated the potential of using machine learning algorithms to address mental health problems, identify these disorders at an early or progenitor stage when intervention may be more effective, and personalize treatment regarding an individual's unique characteristics. However, caution is required to avoid over-interpreting preliminary results, and more work should be done to bridge the gap between mental health research and artificial intelligence in clinical care [24]. Laacke et al. introduced the (ethical) discussion of artificial intelligence in medicine, with reference to mental health. Two AIDD models using social media data and different usage scenarios were proposed, and the extended concept of health-related digital autonomy was proposed [26]. Adikari et al. proposed a comprehensive framework for emotion modeling and analysis under self-structured artificial intelligence. The framework systematically integrates modeling capabilities for unstructured and untagged social media data at a granular level. The validity and effectiveness of the application in real social media environment further proves the methodological novelty of this set of self-organizing artificial intelligence for deep emotions [27]. The above research studies show that artificial intelligence technology can play a very good auxiliary role in the field of mental health, effectively reduce the mental stress, and enhance the ability to cope with difficulties.

## 3. Methods and Design

### 3.1. Emotion Recognition Algorithm Based on Deep Learning

DCNN is featured with a convolutional neural network (CNN) structure with discriminative deep structure and is considered to be the first deep network learning algorithm [28]. CNN imitates the response of biological neurons to external stimuli to achieve the image recognition [29]. The convolutional layer is the feature extraction layer of CNN, and the features of the original input data are extracted through the convolution operation of this layer. The input image or the output of the pooling layer is adjusted by the bias function, and the nonlinear expression of the network is enhanced by the activation function, which can be expressed as

(1) $$\begin{array}{c} {ConvLay}_{j}^{l}=g\left(\underset{i\in M_{j}}{\sum }I_{i}^{l-1}⊗W_{ij}^{l}+B_{j}^{l}\right)\text{,} \end{array}$$

where *g()* represents the activation function, *I*_*i*_^*l*−1^ is the input of the *l-1*^*th*^ layer to the *j*^*th*^ neuron, *W*_*ij*_^*l*^ refers to the convolution kernel of the connected neurons between the *l-1*^*th*^ layer and the *l*^*th*^ layer, ⊗ refers to the convolution operation among connected neurons, and *B*_*j*_^*l*^ represents the offset parameter amount. After the convolution operation in convolution layer, dimension of the feature is very large. When the learned features are directly used to train the classifier in the system, it can cause over-fitting of the model, so that generalization ability of the model is low [30]. Therefore, dimensionality of the feature is reduced through downsampling after the convolutional layer to inhibit the over-fitting.

At present, this algorithm has the following advantages:

1. *Preweighting*. The high-frequency part of music signal generally has low energy, so it is necessary to filter out the low-frequency interference to obtain the high-frequency spectrum more easily.
2. *Framing*. Music signal is a kind of nonstationary signal, but due to the related reasons of human vocal organs, music signal has the nature of being short-term stationary. Therefore, it needs to cut the audio into relatively smooth segments. The length of the cut should not only ensure the stability of the fragment but also be easy to handle. Music signals often appear to be some large peak signals, so the signal is not smooth enough to process. Hence, it is necessary to add windows to the frame signal to smooth the signal.
3. *Mute Frame Detection*. Sometimes there are mute frames without signal parameters in music signal, which affect the result of recognition. A threshold based on the short time energy of the audio signal can be set in this section to detect silent frames. If the energy is below the set threshold, it will be removed.
4. *Feature Extraction*. The quality of extracted feature is directly related to the output result. Features are one of the most used features in acoustic tasks. The human cochlea acts as a set of filters that distinguish between different sounds by filtering characteristics such as frequency bands and bandwidths. Traits mimic this ability.

At the same time, there are some drawbacks, such as the high dimension of the original time-domain features, as well as a lot of redundancy and noise. Therefore, it is necessary to reduce the dimension of the input training data. Principal component analysis is adopted. Principal component analysis is a multivariate statistical method to investigate the correlation between multiple variables. It explores how to reveal the internal structure of multiple variables through a few principal components.

Moreover, when the number of samples is very small, the feature performance extracted by using features is not as good as general features. This also confirms that the deep learning network needs a certain number of samples for training in order to better fit the test samples. Due to the problem of parameter setting, its accuracy has not been greatly improved.

### 3.2. Feature Extraction of Mental Health Data

During analysis of the mental health of college students, the more the analysis items, the more they truly reflect the mental health of the student [31]. In order to reduce the amount of calculation, support vector machine algorithm is adopted to reduce the dimensions of many extracted mental health data of college students. Support vector machine is a learning model that analyzes data and recognizes patterns. It is based on the statistical learning related theories and is particularly good at processing small samples of nonlinear data [32]. The core of the support vector machine lies in the kernel function, and it can convert the samples in the low-dimensional space nonlinearly into the high-dimensional space by mapping the kernel function. The optimal classification function of the support vector machine is defined as follows:

(2) $$\begin{array}{c} f\left(x\right)=sgn\left(\overset{n}{\underset{i=1}{\sum }}\alpha_{i}y_{i}K\left(x_{i},x\right)+b\right)\text{.} \end{array}$$

The main steps for support vector machine to realize model classification are given as follows: input data of the support vector machine are defined, a set of training samples *x*_*i*_ and *y*_*i*_ are given (of which, *y*_*i*_ refers to the type of training sample *x*_*i*_), maximum value of the function is calculated, and *α*_*i*_ is obtained. Such process has to meet the following constraints:

(3) $$\begin{array}{c} \overset{n}{\underset{i=1}{\sum }}\alpha_{i}y_{i}=0,0\leq \alpha_{i}\leq C,i=1,2\ldots n\text{.} \end{array}$$

Function *Q*(*α*) can be calculated with the following equation:

(4) $$\begin{array}{c} Q\left(\alpha \right)=\overset{n}{\underset{i=1}{\sum }}\alpha_{i}-\frac{1}{2}\overset{n}{\underset{i,j=1}{\sum }}\alpha_{i}\alpha_{j}y_{i}y_{j}K\left(x_{i},x_{j}\right)\text{.} \end{array}$$

Then, *W* and *b* can be calculated as follows:

(5) $$\begin{array}{c} W=\overset{n}{\underset{i=1}{\sum }}\alpha_{i}y_{i}x_{i}\text{,}\\ b=\frac{1}{y}-W\cdot x_{s}\text{.} \end{array}$$

In the above equations, *x*_*s*_ is a specific support vector, a certain kernel function *K(x,x*_*i*_) is selected to calculate the optimal classification function of the support vector machine for the test sample *x*, and the calculation result is +1 or −1, which can be taken as the basis for category judgment.

### 3.3. Mental Treatment Based on Artificial Intelligence

**Figure 1:**
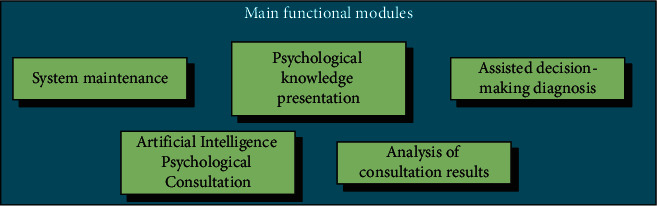
Main functional modules.

Based on the analysis of the mental health of physical education students, an artificial intelligence mental consultation platform is established. The main functional modules to be implemented include system maintenance, mental knowledge presentation, auxiliary decision-making diagnosis, artificial intelligence mental consultation, and consultation result analysis (Figure 1).

**Figure 2:**
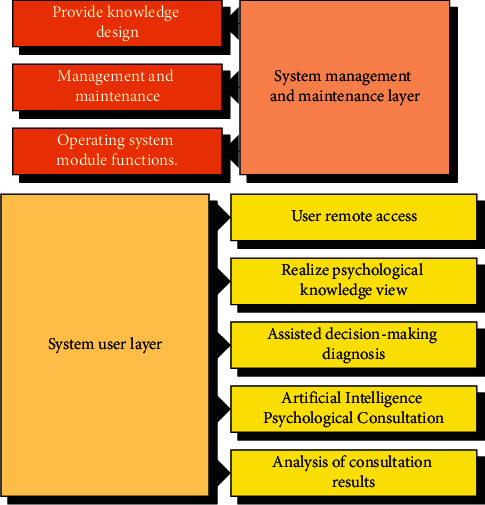
Hierarchy function diagram.

The functional structure is divided into two layers: system management and maintenance layer and system user layer. The system management and maintenance layer refers to the management and maintenance on the server side. It is mainly designed for knowledge engineers and system maintenance personnel, who can operate all module functions of the system. The system user layer is mainly for remote access of ordinary users, who can only realize the mental knowledge view, auxiliary decision-making diagnosis, artificial intelligence mental consultation, and consultation result analysis functions (Figure 2).

### 3.4. Data Training and Parameter Optimization

**Figure 3:**
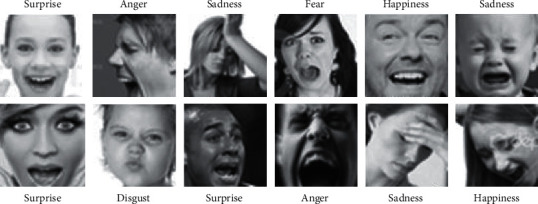
Some samples from Fer-2013.

*Data Source*. The dataset applied in this study is the Kaggle facial emotion recognition dataset (Fer-2013), which was released by Pieere and Aaron at the ICML2013 seminar and consisted of 35,887 facial emotion pictures. There are 4,953, 547, 5,121, 8,989, 6,077, 4,002, and 6,198 pictures on angry, disgust, fear, happiness, sadness, surprise, and neutral emotions [33]. The dataset consists of three parts: the training set contains 28,709 pictures, the validating set contains 3,589 pictures, and the testing set contains 3,589 pictures. Part of the dataset is shown in Figure 3.

The Model training Caffe's framework is adopted in the experiment, the operating system is Ubuntu16.04, the memory is 8G, the central processing unit (CPU) is Intel Core i5-4590@3.30 GHz × 4, and the graphics processing unit (GPU) is GTX1050Ti. The training learning rate is 0.001, momentum is 0.9, Test_interval is 10,000, and the learning strategy is stochastic gradient descent (SGD). Facial expression recognition has related concepts in computer and psychology. In the field of computer science and technology, emotion recognition on account of emotion computing is an important part of the emotion computing program, and the individual's emotional and emotional state can be judged according to the research results of the above content. In the field of psychology, facial emotion recognition refers to the recognition of the specific facial emotional state of the observed object, thereby determining the psychological emotional state of the recognized object currently. For facial emotion recognition, the operational definition of facial emotion recognition is as follows. The recognition responses of individuals to face pictures given in the experiment include response speed and response accuracy. The pictures with obvious emotional characteristics in the standard emotion library are displayed, including positive emotions (smile), negative emotions (sad and scared), and neutral emotions (blank expression), and then subjects are asked to identify the emotional pictures and press the corresponding response button at the same time. The difference of emotion recognition is measured by individual recognition response time and accuracy.

Due to the technological changes brought about by the emergence of computers, the research on facial emotion recognition technology began to emerge and prevail in recent decades. In the process of cognitive science research, due to the diversity of facial expressions and the complexity of the range involved, it has been difficult to identify emotions in the past, and it is extremely difficult to study emotions, especially facial emotions. Therefore, compared with other works involving biology and psychology, the development of emotion recognition is relatively slow. Its results not only are few but also involve only limited applications.

In the influencing factors of facial emotion recognition, the influence of facial muscle cues on emotion recognition has been mentioned. In this experiment, a body cognition-based experimental paradigm is utilized, and facial manipulation is used to influence the perception of emotion. It is found that when they held the pen in their mouth, they rated the cartoons funnier. Other works, such as using emotional understanding of visual images as a dependent variable, have used the same face manipulation paradigm. The results show that facial manipulation can significantly affect emotional understanding of images. After comparison of the response times, it is proved that the response time of the positive picture is shorter than that of the negative picture when biting the pen triggered the smile expression. There is no significant difference in response time between the two images when suppressing the smiling expression.

*Parameter Optimization*. The sample dataset is kept fixed in the experiment. The mental health data structure of each student is composed of a one-dimensional column vector of 11 elements, and the maximum dimension is 11, so the range of d can be determined as [2, 10], and the step size is 1; the value interval of the neighbor parameter *K* is [3, 10], and the step size is 1. In the experiment, SVM is undertaken as the classifier, and its kernel function is class support vector classification (C-SVC). The specific values of the parameters involved are degree = 3, gamma = 0.5, coef_0_ = 0, and penalty factor *C* = 1.

### 3.5. Performance Evaluation and Interactive Analysis

*Performance Evaluation*. In mental health prediction, the most common algorithms are BPNN and DT algorithm [34], while locally linear embedding (LLE) algorithm is recently published in many articles with the ACC as high as 92% [35]. For this reason, different algorithms are applied for comparative analysis. In order to analyze the performance of the model more directly, ACC is adopted to represent the accuracy of the model in the testing set, and F1 is undertaken as the comprehensive performance evaluation index of the model, which is the harmonic mean of the ACC and the recall (Rec) [36]. The specific equation is as follows:

(6) $$\begin{array}{c} ACC=\frac{\sum_{i=1}^{s}Tp_{i}}{\sum_{i=1}^{s}\left(Tp_{i}+{FN}_{i}\right)}\text{,} \end{array}$$

(7) $$\begin{array}{c} F_{1}=\frac{2*Pre*Rec}{Pre+Rec}\text{.} \end{array}$$

The facial emotions in the testing set are divided into categories, *Tp*_*i*_ is used to represent the number of correctly recognized emotions in a category, and FN_*i*_ represents the number of incorrectly recognized emotions in this category [37–39]. In (7), *Pre* refers to the precision, which means the proportion of all samples classified as positive, which are correctly classified, and *Rec* refers to the recall, which means the proportion of samples classified as positive in the actual positive samples.

*Interactive Analysis*. 120 students majoring in physical education in a university are selected and divided into two groups. One group is named as the experimental group (using an interactive system for teaching), and the other group is named as the control group (with normal mental counseling), which is accepted with a three-day mental counseling. When the experiment is over, questionnaires are distributed and collected to evaluate the two teaching methods [40–43]. The questionnaires are based on online questionnaires, covering three aspects: the effect, role, and acceptance of artificial intelligence mental counseling. 110 questionnaires are received, including 105 valid questionnaires. Then, the data are tested with the reliability and validity analysis, correlation analysis, and hypothesis test, so as to obtain the analysis results of the effect of human-computer interaction psychotherapy. The evaluation results of validity and reliability of the questionnaire are shown in Table 1.

Standardized questionnaires must be tested for reliability and validity before they are used. If small sample surveys are used to predict target groups, they have higher requirements to produce questionnaires or they are prone to statistical errors. According to the analysis in Table 1, the validity evaluation results of the questionnaire are good, so it can be used in the mental health investigation of college students.

## 4. Results and Analysis

### 4.1. Comparison of Facial Emotion Recognition Feature Algorithms

**Figure 4:**
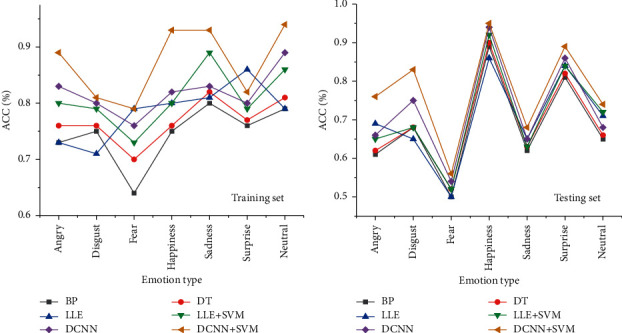
Comparison results of facial emotion recognition feature algorithms. (a) The numerical results of training. (b) The numerical results of testing.

As shown in Figure 4, different algorithm models show greater differences in the training set and small differences in the testing set. The traditional backpropagation neural network (BPNN) and decision tree (DT) algorithm have the worst performance in the early warning of mental health for college students. After the locally linear embedding (LLE) algorithm with local linear embedding is adopted, performance of the model has been improved with an average improved adaptive cruise control (ACC) of 2%. When the SVM algorithm is added, the average ACC has increased by 3.47%. DCNN algorithm has the best performance with an average ACC of 81.86%. After the SVM classification is added, the performance of the algorithm has been greatly improved, so that the highest ACC can reach 94% with an average ACC of 87.28%. The above results show that the deep learning significantly improves the ACC of model recognition through data mining compared with traditional algorithms, and the SVM classification performance can complement the over-fitting of DCNN, thereby significantly improving the ACC of the model.

### 4.2. Determination of Optimal Parameters for Early Warning of Mental Health

**Figure 5:**
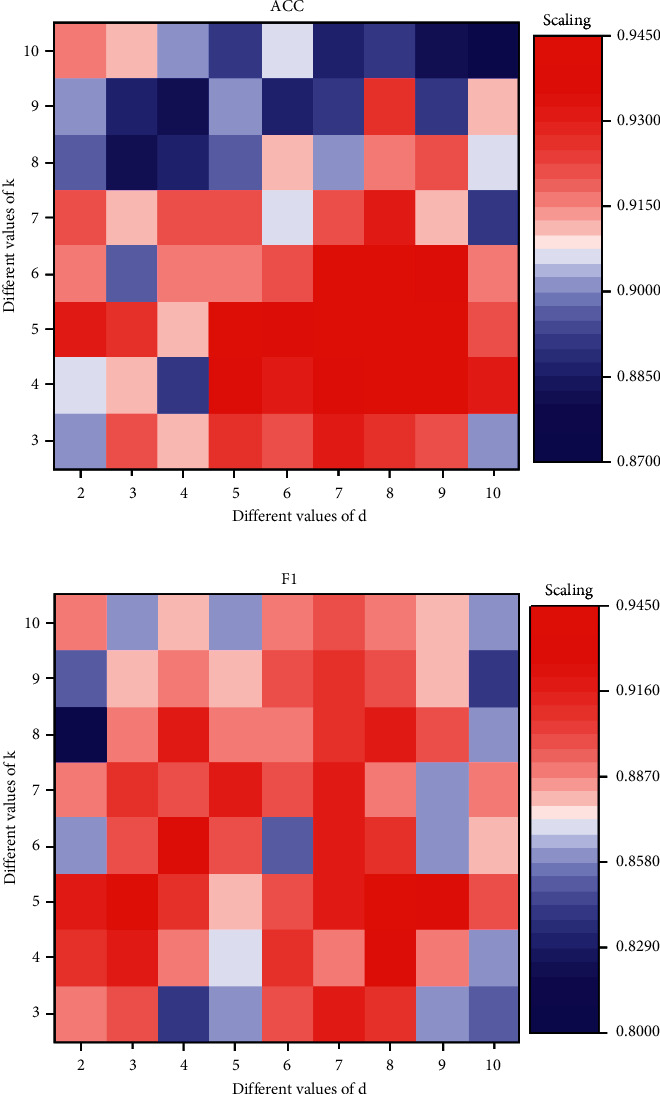
Determination of optimal parameters of early warning model of mental health. (a) The numerical results of training. (b) The numerical results of testing.

As shown in Figure 5, if *k* is too small, it will affect the global performance of the model, and if *k* is too large, the original nonlinear characteristics will not exist after the data are reduced. The data in the status space may overlap if d is too small, and the noise will be introduced in the reduced dimensionality dataset so that the ACC is decreased if d is too large. It is found that the highest ACC of the model is 94.5% when *k* is 5 and *d* is 8, so they are applied for model training and early warning in subsequent experiments so as to accurately assess the mental health of physical education students.

### 4.3. Psychotherapy Effect of Human-Computer Interaction System

**Figure 6:**
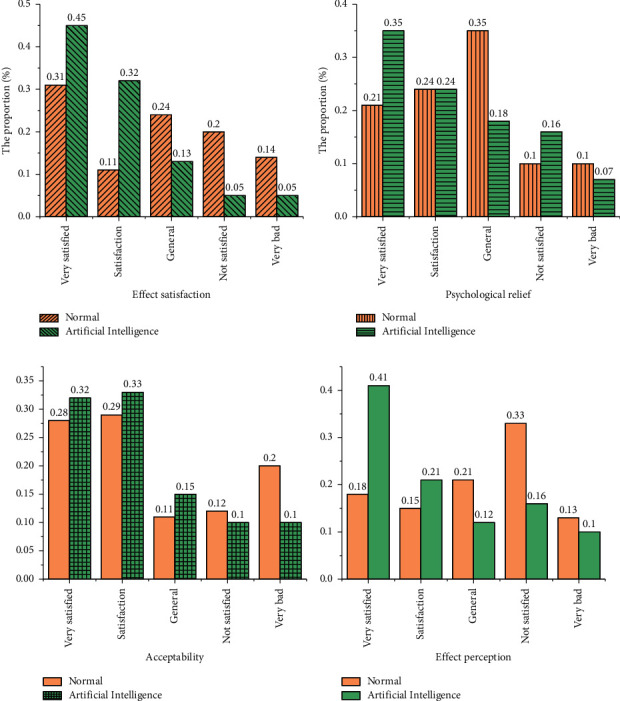
Psychotherapy effect analysis of human-computer interaction system. (a) The result of effect satisfaction. (b) The result of psychological relief. (c) The result of acceptability. (d) The result of effect perception.

Figure 6 suggests that most of the students are quite satisfied with the artificial intelligence mental counseling, and the proportion of “very satisfied” is the highest (45%). The satisfaction to the artificial intelligence method is obviously higher than that of professional teachers. General mental questions are related to privacy, and the application of human-computer interaction can solve it effectively. On the other hand, human-computer interaction is more targeted and can effectively analyze the mental problems of physical education students. Compared with the traditional way, 30% of students think that the human-computer interaction is acceptable. After mental counseling with artificial intelligence, 41% of students think that they are more comfortable mentally, while only 18% of students think the traditional way of mental counseling is more comfortable. Based on the above results, it is found that the use of artificial intelligence for psychotherapy for physical education students is more effective, and this method is also widely accepted by students. This system plays a good auxiliary role in daily learning and making friends of physical education students.

## 5. Discussion

By using different algorithms, it is found that the performance of the model can be significantly affected. SVM and DCNN algorithms can achieve the highest performance of the model. It is also found that different values of *k* and d will affect the data in space, resulting in data overlap. Hence, the data noise is too large, affecting ACC value. After the questionnaire is utilized to investigate the psychological status of the students, it is found that the satisfaction of the artificial intelligence method is significantly higher than that of professional teachers. General psychological problems are related to privacy, and the application of human-computer interaction can be effectively solved. Moreover, human-computer interaction is more targeted and can effectively analyze the psychological problems of sports students.

## 6. Conclusion

Based on the analysis of the current research status of mental health and the construction of mental early warning systems for college students, an emotional recognition system with higher recognition ACC is established for physical education students through deep learning of the face database. Compared with other algorithm models, the established system has the ability of learning and analysis. Through the optimal analysis of parameters, its mental health prediction performance has reached a better state. With the help of artificial intelligence technology, the automatic early warning treatment system of mental health teaching has achieved better practical results. An automatic early warning platform for mental health education is constructed for physical education students, but there are still many shortcomings in the study. Firstly, it considers the effect of activation function and model structure on the performance and optimizes the main parameters, but the other parts of the current neural network can significantly improve the prediction performance of model. Secondly, it can consider using data mining technology for a more in-depth analysis of the acquired face data to improve the consulting effect of human-computer interaction and establish different communication methods for different emotions to avoid more mechanized communication. Mechanized communication is a type of queuing system utilized to describe the communication recovery process of mechanized repair units. The working algorithm of the simulation model of the recovery process of the communication means of the mechanized braiding unit is given. Therefore, how to better improve the performance and calculation accuracy of the model will be the main research objective in the subsequent research work. In the follow-up, further analysis and development will focus on these two aspects, so as to provide more effective diagnosis and treatment systems for college students of different majors.

## Figures and Tables

**Table 1:** Questionnaire validity evaluation.

Content	Level		
	Very high (%)	High (%)	Low (%)
Content evaluation	45	52	3
Quantity evaluation	39	60	1
Structure evaluation	26	59	15

## Data Availability

The raw data supporting the conclusions of this article will be made available by the authors, without undue reservation.

## References

[1] Gallie D. (2019). Research on work values in a changing economic and social context. *The Annals of the American Academy of Political and Social Science*.

[2] Vela J. C., Smith W. D., Whittenberg J. F., Guardiola R., Savage M. (2018). Positive psychology factors as predictors of Latina/o college students’ psychological grit. *Journal of Multicultural Counseling and Development*.

[3] Kassymova Tokar G. . К., Tashcheva O. V., Slepukhina A. I. (2019). Impact of stress on creative human resources and psychological counseling in crises. *J. International journal of education and information technologies*.

[4] Song Y. (2019). Gratifications for social media use in entrepreneurship courses: learners’ perspective. *Frontiers in Psychology*.

[5] Oduaran C., Okorie N. (2018). Community radio, family and psychological support for sexual harassment issues: a study of Yoruba usage. *J. Media Watch.*.

[6] Ahmad S. I., Leventhal B. L., Nielsen B. N., Hinshaw S. P. (2020). Reducing mental-illness stigma via high school clubs: a matched-pair, cluster-randomized trial. *Stigma and Health*.

[7] Yang R., You X., Zhang Y., Lian L., Feng W. (2019). Teachers’ mental health becoming worse: the case of China. *International Journal of Educational Development*.

[8] Kalkbrenner M., Hernández T. J. (2017). Community college students’ awareness of risk factors for mental health problems and referrals to facilitative and debilitative resources. *Community College Journal of Research and Practice*.

[9] Chen M. (2019). The impact of expatriates’ cross-cultural adjustment on work stress and job involvement in the high-tech industry. *Frontiers in Psychology*.

[10] Wang Y., Duan Z., Ma Z. (2020). Epidemiology of mental health problems among patients with cancer during COVID-19 pandemic. *Translational Psychiatry*.

[11] Hong W., Liu R. D., Ding Y., Fu X., Zhen R., Sheng X. (2021). Social media exposure and college students’ mental health during the outbreak of CoViD-19: the mediating role of rumination and the moderating role of mindfulness. *Cyberpsychology, Behavior, and Social Networking*.

[12] Wang J., Zhang Z., Luo H., Liu Y., Chen W., Wei G. (2019). Research on early warning model of college students’ psychological crisis based on genetic BP neural network. *American Journal of Applied Psychology*.

[13] Huhtiniemi M., Sääkslahti A., Watt A., Jaakkola T. (2019). Associations among basic psychological needs, motivation and enjoyment within Finnish physical education students. *Journal of sports science & medicine*.

[14] Liu F., Zhou N., Cao H. (2017). Chinese college freshmen’s mental health problems and their subsequent help-seeking behaviors: a cohort design (2005-2011). *PLoS One*.

[15] Kaplan A., Haenlein M. (2019). In my hand: who’s the fairest in the land? On the interpretations, illustrations, and implications of artificial intelligence. *Business Horizons*.

[16] Shah N., Irani Z., Sharif A. M. (2017). Big data in an HR context: exploring organizational change readiness, employee attitudes and behaviors. *Journal of Business Research*.

[17] Kulesza M., Odrowaz-Coates A., Perkowska-Klejman A. (2021). Suicide rates amongst adolescents A mental health practitioner’s perspective in Poland and a global, Big Data context. *Resocjalizacja Polska*.

[18] Huang F., Ding H., Liu Z. (2020). How fear and collectivism influence public’s preventive intention towards COVID-19 infection: a study based on big data from the social media. *BMC Public Health*.

[19] Simon G. E. (2019). Big data from health records in mental health care: hardly clairvoyant but already useful. *JAMA Psychiatry*.

[20] Park S. H., Han K. (2018). Methodologic guide for evaluating clinical performance and effect of artificial intelligence technology for medical diagnosis and prediction. *Radiology*.

[21] Park S. H., Kressel H. Y. (2018). Connecting technological innovation in artificial intelligence to real-world medical practice through rigorous clinical validation: what peer-reviewed medical journals could do. *Journal of Korean Medical Science*.

[22] Wu W., Wang H., Zheng C., Wu Y. J. (2019). Effect of narcissism, psychopathy, and machiavellianism on entrepreneurial intention—the mediating of entrepreneurial self-efficacy. *Frontiers in Psychology*.

[23] D’Alfonso S., Santesteban-Echarri O., Rice S. (2017). Artificial intelligence-assisted online social therapy for youth mental health. *Frontiers in Psychology*.

[24] Graham S., Depp C., Lee E. E. (2019). Artificial intelligence for mental health and mental illnesses: an overview. *Current Psychiatry Reports*.

[25] Kalmady S. V., Greiner R., Agrawal R. (2019). Towards artificial intelligence in mental health by improving schizophrenia prediction with multiple brain parcellation ensemble-learning. *Npj Schizophrenia*.

[26] Laacke S., Mueller R., Schomerus G., Salloch S. (2021). Artificial intelligence, social media and depression. A new concept of health-related digital autonomy. *The American Journal of Bioethics*.

[27] Adikari A., Gamage G., de Silva D., Mills N., Wong S. M. J., Alahakoon D. (2021). A self structuring artificial intelligence framework for deep emotions modeling and analysis on the social web. *Future Generation Computer Systems*.

[28] Jin K. H., McCann M. T., Froustey E., Unser M. (2017). Deep convolutional neural network for inverse problems in imaging. *IEEE Transactions on Image Processing*.

[29] Liang H., Li Q. (2016). Hyperspectral imagery classification using sparse representations of convolutional neural network features. *Remote Sensing*.

[30] Xu Q., Zhang M., Gu Z., Pan G. (2019). Overfitting remedy by sparsifying regularization on fully-connected layers of CNNs. *Neurocomputing*.

[31] Rogoza R., Żemojtel-Piotrowska M., Kwiatkowska M. M., Kwiatkowska K. (2018). The bright, the dark, and the blue face of narcissism: the Spectrum of narcissism in its relations to the metatraits of personality, self-esteem, and the nomological network of shyness, loneliness, and empathy. *Frontiers in Psychology*.

[32] Huang S., Cai N., Pacheco P. P., Narrandes S., Wang Y., Xu W. (2018). Applications of support vector machine (SVM) learning in cancer genomics. *CANCER GENOMICS and PROTEOMICS*.

[33] Gautam K. S., Thangavel S. K. (2019). Video analytics-based facial emotion recognition system for smart buildings. *International Journal of Computers and Applications*.

[34] Feng X. L., Gao J. (2018). Attendance data analysis with decision regression tree on spark. *Journal of Inner Mongolia University of Technology (Natural Science Edition)*.

[35] Vella S. A., Swann C., Batterham M. (2018). Ahead of the game protocol: a multi-component, community sport-based program targeting prevention, promotion and early intervention for mental health among adolescent males. *BMC Public Health*.

[36] Chicco D., Jurman G. (2020). The advantages of the Matthews correlation coefficient (MCC) over F1 score and accuracy in binary classification evaluation. *BMC Genomics*.

[37] Chen J. J., Jiang T. N., Liu M. F. (2021). Family socioeconomic status and learning engagement in Chinese adolescents: the multiple mediating roles of resilience and future orientation. *Frontiers in Psychology*.

[38] Liu K., Ke F., Huang X. (2021). DeepBAN: a temporal convolution-based communication framework for dynamic WBANs. *IEEE Transactions on Communications*.

[39] Meng F., Xiao X., Wang J. (2022). Rating the crisis of online public opinion using a multi-level index system. *The International Arab Journal of Information Technology*.

[40] Zhang Z., Tian J., Huang W., Yin L., Zheng W., Liu S. (2021). A haze prediction method based on one-dimensional convolutional neural network. *Atmosphere*.

[41] Shang K., Chen Z., Liu Z. (2021). Haze prediction model using deep recurrent neural network. *Atmosphere*.

[42] Liu Y., Tian J., Hu R. (2022). Improved feature point pair purification algorithm based on SIFT during endoscope image stitching. *Frontiers in Neurorobotics*.

[43] Choi Y., Wang J., Zhu Y., Lai W. F. (2021). Students’ perception and expectation towards pharmacy education: a qualitative study of pharmacy students in a developing country. *Indian Journal of Pharmaceutical Education and Research*.

